# Macula Characteristics in Young Saudi Male Patients with Myopia: A Comparative Hospital-Based Study

**DOI:** 10.7759/cureus.69168

**Published:** 2024-09-11

**Authors:** Raghda F. Mutwaly, Abdelaziz M. Elmadina, Saif H. Alrasheed, Majid A. Moafa, Sulaiman Aldakhil

**Affiliations:** 1 Department of Optometry, College of Applied Medical Sciences, Qassim University, Buraydah, SAU

**Keywords:** risk factor, young adult, macula thickness, optical coherence tomography, myopia

## Abstract

Background: Myopia is associated with changes in the posterior segment of the eye, including the macula, which may contribute to potential blindness. Therefore, the study aims to evaluate the macula integrity in young myopic patients through optical coherence tomography (OCT).

Methods: A cross-sectional study was conducted at Qassim University optometry clinics from January to June 2024, involving 30 healthy young males with myopia and 30 with emmetropia. Clinical examination and OCT imaging were used to gather data on macular volume and thickness. The results were analyzed using descriptive statistics and correlation analysis.

Results: The study included 60 myopic eyes and 60 emmetropic eyes. The participants' mean age was 22.10 ± 1.65 years (p = 0.135). A significant difference was found between myopic and emmetropic eyes in fovea, parafovea, perifovea, and total macula thickness and volume (P < 0.05). Additionally, the study showed a significant positive correlation between the magnitude of myopia and fovea thickness (r = 0.297, p = 0.011) and negative correlations with perifovea thickness (r = -0.418, p < 0.001), total macula thickness (r = -0.353, p = 0.003), and total macula volume (r = -0.352, p = 0.003). However, parafoveal thickness had no significant correlation with the severity of myopia (r= -0.107; p=0.207). Fovea thickness was higher in high myopia (255.40 ± 23.51 µm) compared to low (238.69 ± 15.98 µm) and moderate (248.15 ± 8.70 µm) myopia. Perifovea thickness, macula thickness, and macula volume were significantly lower in high myopia (p ˃ 0.05).

Conclusion: Myopia influences macular parameters compared to emmetropia. It increases fovea thickness and decreases total macula thickness and volume as well as perifovea thickness. High myopia has more alterations in macula parameters.

## Introduction

Myopia is a common refractive error that leads to visual impairment. Uncorrected myopia results in the inability of individuals to see distant objects clearly [1,2]. Additionally, myopia makes changes in the posterior eye segment, including macula that may result in potential blindness [3,4]. Assessment of the retinal layer’s thickness, such as ganglion cell layer, retinal nerve fiber layer, and macula thickness, is essential in early detection and diagnosis of glaucoma. It is also helpful in the diagnosis of some neurological diseases such as increased intracranial pressure [5]. Many studies have investigated the effect of refraction on the posterior segment of the eye, particularly the ganglion cell layer, retinal nerve fiber layer, and macular parameters. However, there is a lack of studies conducted in Saudi Arabia.

Previous studies investigated the impacts of myopia on retinal thickness measurements at different regions in healthy eyes. They measured all macular parameters such as total macula thickness, total macula volume, central (fovea) thickness (in 1 mm zone), inner circle (parafovea) (in 1-3 mm zone), and outer circle (perifovea) (in 3-6 mm zone) thickness. They found that the parafovea (outer circle) is significantly thicker than the perifovea (inner circle) in emmetropic and myopic eyes [6,7]. However, fovea thickness was higher in myopic eyes compared to emmetropic eyes [8]. Studies by Ooto et al. in Japan [9] and Zhao et al. in China [10] showed that the fovea is thinner in moderate myopia than in high myopia. A study by Liu et al. showed that the fovea is thicker in high myopia [11].

Fovea thickness is positively correlated with axial length and increases as myopia progresses [12]. A study also demonstrated that the macula was thicker in men than in women [13]. In addition, parafovea and perifovea were thicker in emmetropic eyes than in moderate-to-high myopic eyes but were identical to those in eyes with low myopia [13,14]. Additionally, the inner regions (parafovea) had a negative correlation with axial length as myopia progresses [13]. A study by Zereid and Osuagwu in Riyadh, Saudi Arabia, showed that parafoveal and perifoveal regions were thinner in moderate to high myopic eyes than in non-myopic eyes [15]. However, a study by Cheng et al. in a Chinese population with high myopia reported that parafovea thickness decreases with the progression of myopia [16]. Other studies showed that parafovea was negatively associated with the magnitude of myopia and pathologic myopia had severe decreases of the parafovea [17,18]. The overall thickness of the macula is negatively associated with axial eye length. Also, perifovea thickness decreased with the progression of myopia [19-21]. In three studies in Indian and Japanese populations, perifovea has been found to be thicker in low-to-moderate myopia compared to high myopia [22-24]. However, studies conducted in India by Malakar et al. [25] and Hwang and Kim [26] in China reported that the perifovea has a negative correlation with myopia progression.

Previous studies on different ethnicities have shown various macular alterations in myopic eyes. Therefore, the current study was conducted to evaluate macula parameters in young Saudi males using optical coherence tomography. The assessment included total macula thickness, total macula volume as well as fovea, perifovea, and parafovea thickness.

## Materials and methods

A cross-sectional comparative hospital-based study was conducted on 30 healthy young male subjects with myopia and 30 with emmetropia (60 myopic eyes and 60 emmetropic eyes), at optometry clinics at the Department of Optometry,, College of Applied Medical Sciences, Qassim University, Buraydah, Saudi Arabia, from February to June 2024. The study was approved by the Committee of Research Ethics, Deanship of Graduate Studies and Scientific Research, Qassim University (approval number: 24-02-09) and followed the Declaration of Helsinki. Informed consent was obtained from each participant before conducting clinical examinations.

Inclusion and exclusion criteria

The study included young adult males aged 18-25 years with healthy eyes, classified as myopic (SE≤-1.00DS) or emmetropic (SE ≤ 0.50DS), with normal intraocular pressure and wearing spectacles only. Individuals with a history of ocular or systemic diseases, intraocular surgery, refractive surgery, eye trauma, or neurological diseases were excluded from the study.

Sample size

The study used a convenient sampling procedure to select data.

Data collection procedures

Data was collected through clinical examination, including demographic information (age and gender), medical history, and assessment of vision via projector vision chart (decimal notation) and refraction via auto ref/keratometer (RK 4800, Topcon Corporation, Tokyo, Japan). Macular parameters were measured using a three-dimensional (3D) optical coherence tomographer (OCT) using macular cube scan protocol. The measurements included total macula thickness (TMT), total macula volume (TMV), central subfield (fovea) thickness, inner circle (parafovea) thickness, and outer circle (perifovea) thickness. Parafovea and perifovea thickness were important to measure due to different anatomical features at the macula surface. It was measured in four zones including superior, inferior, temporal, and nasal parts. Then the average thickness was obtained. Myopic groups were classified into low (≤ -1.00 to ˃ -3.00D), moderate (˂-3.00 to ˃-6.00D), and high (high (≤ -6.00D) myopia. Three measurements were taken for each variable and the average was obtained.

Statistical analysis

The study utilized IBM SPSS Statistics for Windows, Version 25.0 (Released 2017; IBM Corp., Armonk, New York, United States) for data analysis, obtaining descriptive statistics, and finding correlation at a 95% confidence interval (CI). An independent sample t-test was used to find the correlation between the demographic and macula parameters in myopic and emmetropic eyes. Pearson’s correlation test was used to find the correlation between the magnitude of myopia and macula parameters. The normality of variance was checked by the Kolmogorov-Smirnov test. The analysis of variance (ANOVA) test was used to find the correlations between macula parameters among low, moderate, and high myopia. A p-value < 0.05 was considered to be significant.

## Results

A total of 60 healthy male subjects (120 eyes) were included in this study. Thirty participants (60 eyes) were myopic and 30 (60 eyes) were emmetropic. The age of the myopia group had a range of 18-25 years (mean 22.10±1.65 years) and the age of the emmetropia group had a range of 18-26 years (mean 22.13±1.62 years) (p=0.135). In addition, an independent sample t-test was used to find the correlation between the macula parameters in myopic and emmetropic eyes. There was a significant difference in fovea thickness between myopic (243.23±15.58 μm) and emmetropic (233.07±19.501 μm) eyes (p = 0.002). A significant difference in parafovea thickness was found between myopic (310.62±9.49 μm) and emmetropic (311.08±14.36 μm) eyes (p = 0.044), but no difference was observed in its superior, inferior, nasal, and temporal parts (p > 0.05). However, total perifovea thickness was significantly different between myopic (264.49±10.05 μm) and emmetropic (272.30±12.90 μm) eyes (p = 0.002). All perifovea parts (superior, inferior, nasal, and temporal) were significantly different between myopic and emmetropic eyes (p < 0.05). Total macula thickness was significantly different between myopic (274.14±9.05 μm) and emmetropic (279.38±12.20 μm) eyes (p = 0.020), as well as total macula volume between myopic (7.75±0.26 mm³) and emmetropic (6.96-8.56 mm³) eyes (p = 0.020). (Table 1).

**Table 1:** Demographic and clinical parameters among myopic and emmetropic eyes ^a^ Independent sample t-test, CI 95%, sig. level 0.05. SE: spherical equivalent; FT: fovea thickness; PAFT: parafovea thickness; SPAFT: superior parafovea thickness; IPAFT: inferior parafovea thickness; TPAFT: temporal parafovea thickness; NPAFT: nasal parafovea thickness; PEFT: perifovea thickness; SPEFT: superior perifovea thickness; IPEFT: inferior perifovea thickness; TPEFT: temporal perifovea thickness; NPEFT: nasal perifovea thickness; TMT: total macula thickness; TMV: total macula volume

Variable	Myopia (n = 60)		Emmetropia (n = 60)		P-Value^a^
	Mean±SD	Range	Mean±SD	Range	
Age (years)	22.10±1.65	18 - 25	22.13±1.62	18 - 26	0.135
SE (D)	3.14±2.02	0.75 - 9.25	0.28±0.20	0.00 – 0.50	˃0.001
Vision (decimal)	0.38 ± 0.28	0.02-1.00	0.99±0.06	0.63 – 1.00	0.030
FT (μm)	243.23±15.58	207-285	233.07±19.501	196-282	0.002
PAFT (μm)	310.62±9.49	292-336	311.08±14.36	270-332	0.044
SPAFT (μm)	314.42±10.40	294-342	315.83±14.09	275 – 340	0.550
IPAFT (μm)	312.00±9.89	289-336	313.40±13.85	275 – 336	0.556
NPAFT (μm)	315.58±10.53	295-343	313.23±16.58	263 – 336	0.375
TPAFT (μm)	300.47±9.67	282-324	301.83±14.31	264 – 324	0.559
PEFT(μm)	264.49±10.05	244-286	272.30±12.90	239-303	0.002
SPEFT (μm)	265.97±10.57	244-287	273.13±12.91	239-296	0.004
IPEFT (μm)	256.217±11.96	232-280	266.13±16.70	230-334	0.001
NPEFT (μm)	284.50±12.66	249-314	291.15 ± 14.68	261-328	0.025
TPEFT (μm)	251.28±12.93	232-315	258.77±12.65	224-228	0.003
TMT (μm)	274.14±9.05	258-295	279.38±12.20	246-302	0.020
TMV (mm^3^)	7.75±0.26	7.31-8.34	7.90 ± 0.35	6.96-8.56	0.020

On the other hand, Pearson’s correlation was used to find the effect of the magnitude of myopia on different macular parameters. It demonstrated significant positive correlation with fovea thickness (r=0.297; p=0.011) and negative correlation with perifovea thickness (r=-0.418; p ˂0.001), total macula thickness (r= -0.353; p=0.003) and total macula volume (r= -0.352; p=0.003). However, parafovea thickness had no significant correlation with the severity of myopia (r= -0.107; p=0.207) (Table 2). 

**Table 2:** Correlation between magnitude of myopia and macula parameters ^a^ Pearson’s correlation test, CI 95%, sig. level 0.05.

Parameter	r	P-Value^a^
Vision (decimal)	-0.540	˂0.001
Fovea thickness (μm)	0.297	0.011
Parafovea thickness (μm)	-0.107	0.207
Perifovea thickness (μm)	-0.418	˂0.001
Total macula thickness (μm)	-0.353	0.003
Total macula volume (mm^3^)	-0.352	0.003

Furthermore, ANOVA yielded significant differences in vision, best corrected visual acuity (BCVA), and spherical equivalent (SE) between low (1.77±0.64 D), moderate (4.35±0.90D), and high myopia (7.87±1.51D) (p˂0.05). Furthermore, there was a significant difference between myopia groups in the thickness of fovea (p= 0.004), perifovea (p = 0.025), and total macula (p = 0.009) as well as total macula volume (p = 0.047). However, no significant difference was found in parafovea thickness between myopia groups (p = 0.305) (Table 3). 

**Table 3:** Demographic and macular parameters among myopia groups ^a^ Analysis of variance (ANOVA) test, CI 95%, sig. level 0.05. SE: spherical equivalent; BCVA: best corrected visual acuity

Variable	Low (n=30) (≤ -1.00 to ˃ -3.00D)	Moderate (n=20) (˂ -3.00 to ˃ -6.00D)	High (n=10) (≤ -6.00D)	P-Value^a^
	Mean±SD (range)	Mean±SD (range)	Mean±SD (range)	
Age (years)	22.31±1.47 (19-25)	22.65±1.81 (18 - 24)	22.65±1.81 (20 - 24)	0.090
SE (D)	1.77± 0.64 (0.75-3.00)	4.35±0.90 (3.25 - 6.00)	7.87±1.51 (6.50-9.25)	˂0.001
Vision (decimal)	0.52±0.27 (0.10-1.00)	0.20±0.13 (0.05 – 0.50)	0.08±0.05 (0.02-0.16)	0.040
BCVA (decimal)	1.00±0.00 (1.00-1.20)	1.00 ± 0.00, 1.00	1.00±0.00, 1.00	0.020
Fovea thickness (μm)	238.69±15.98 (207-285)	248.15±8.70 (228-261)	255.40±23.51 (231-279)	0.004
Parafovea thickness (μm)	310.89±8.99 (294-329)	310.10±11.45 292-336	310.75±4.18 (307-316)	0.305
Perifovea thickness (μm)	267.06±10.48 (249-286)	261.08±9.27 (244-272)	260.20±2.75 (256-263)	0.025
Total macula thickness (μm)	275.94±9.32 (260-295)	271.68±9.07 (258-285)	271.34±3.30 (267.9-275.4)	0.009
Total macula volume (mm^3^)	7.80±0.26 (7.35-8.34)	7.68±0.26 (7.31-8.08)	7.67±0.09 (7.58-7.79)	0.047

## Discussion

Myopia is commonly accompanied by alterations in the posterior eye segment, including the macula, which may lead to changes in visual function and potential visual impairment. Therefore, the present study was conducted to assess the integrity of different macula regions in young myopic patients using OCT. To the best of our knowledge, it was the first such study conducted on Saudi males. The findings of the current study are hoped to assist practitioners in the management of myopia by exhibiting the early macula changes in relation to the severity of myopia.

The study showed that there was no significant difference in age between the myopia and emmetropia groups (p = 0.090). A comparison was done of various macular parameters between myopic and emmetropic eyes, shedding light on significant differences. A significantly greater foveal thickness was seen in myopic eyes compared to emmetropic eyes (p = 0.002). This finding may reflect structural changes associated with myopia, potentially related to the elongation of the eyeball and stretching of retinal layers. However, anatomically, the vitreous body is firmly attached to the macula at the foveal area which could result in vitreomacular traction and lead to an increase in foveal thickness [2,5]. This result agrees with previous studies which reported that fovea thickness was higher in myopic eyes compared to emmetropic eyes [7,8,10]. 

The present study also revealed a significant difference in the inner circle thickness (parafovea) between myopic and emmetropic eyes (p = 0.044), consistent with findings from other previous studies [4,9,25]. Furthermore, myopic eyes demonstrated significantly reduced thickness in the outer circle (perifovea) across all quadrants compared to emmetropic eyes (p < 0.05). This finding underscores the impact of myopia on perifoveal structural integrity, potentially contributing to visual disturbances and disease progression. However, the TMT and TMV were significantly lower in myopic eyes compared to emmetropic eyes (p < 0.05). These findings suggest widespread macular thinning associated with myopia, highlighting the importance of monitoring macular health in myopic individuals. These results are consistent with those of previous studies [12,13,15] (Table 1). Correlation between the magnitude of myopia and macula parameters revealed that foveal thickness has a significant positive correlation with the degree of myopia (r = 0.297; p = 0.011). This suggests that as myopia increases, the fovea thickness tends to increase. The parafoveal thickness does not exhibit a significant correlation with myopia progression (r = -0.107, p = 0.207), indicating that changes in myopia degree are not strongly associated with alterations in the parafoveal area [9].

Furthermore, the study demonstrated a significant negative correlation (r = -0.418; p = 0.001) between myopia progression and perifovea thickness. This indicates that as myopia severity increases, perifovea thickness tends to decrease significantly. Additionally, a significant negative correlation was found between myopia and total macula thickness (r = -0.353, p = 0.003) as well as total macula volume (r = -0.352, p = 0.003) (Table 2). This suggests that as myopia increases, total macula thickness and total macula volume tend to decrease. These results are consistent with previous studies, which reported that the parafovea and perifovea regions were thicker in emmetropic eyes compared to myopic eyes [17-19]. In addition, a significant increase was found in fovea thickness (p= 0.004) in high myopic eyes compared to low and moderate myopia. Perifovea (p = 0.025), TMT (p = 0.009), and TMV (p = 0.047) showed a significant decrease in the high myopia group. However, no significant difference was found in parafovea thickness between myopia groups (p = 0.305) (Table 3). These results are consistent with other studies reported that TMT, TMV, and perifovea thickness are lower in high myopic eyes compared to low and moderate myopia [20,21,26].

The observed decrease in perifovea thickness, macula thickness, and macula volume with increasing myopia severity may be attributed to axial elongation and stretching of the retina, leading to the thinning of retinal layers. Conversely, the positive correlation between fovea thickness and myopia severity suggests potential compensatory mechanisms involving vitreous traction and axial elongation. These findings reflect a complex relationship between myopia and retinal morphology, highlighting potential structural macular changes associated with myopia that may impact visual function. The results emphasize the need for further research such as longitudinal studies and exploring interventions to mitigate macula changes in high myopia.

A limitation of the current study is that it may not provide information about the macula characteristics in female myopic subjects. Also, data was selected by a convenient sampling procedure. In contrast, the strength of the study lies in the inclusion of relatively young subjects, ensuring a well-established presence of myopia and avoiding age-related changes.

## Conclusions

The study concluded that myopia influences various macular parameters compared to emmetropia. It increases fovea thickness and decreases total macular thickness, total macular volume and perifovea thickness. High myopia has more alterations in macula parameters than low myopia. Thus, the study emphasizes the implications of OCT examinations in clinical practice and public health in Saudi Arabia in patients with high myopia. 
